# Additional compensation for health care professionals in COVID-19 pandemic: an essay in the light of health law

**DOI:** 10.47626/1679-4435-2022-761

**Published:** 2022-03-30

**Authors:** Dorival Fagundes Cotrim-Junior, Lucas Manoel da Silva Cabral, João Paulo Almeida Brito

**Affiliations:** 1 Departamento de Política, Planejamento e Administração em Saúde, Instituto de Medicina Social Hesio Cordeiro da Universidade do Estado do Rio de Janeiro, Rio de Janeiro, RJ, Brazil.; 2 Departamento de Pós-Graduação em Direito, Pontifícia Universidade Católica do Rio de Janeiro, Rio de Janeiro, RJ, Brazil.; 3 Escola de Governo Fiocruz Brasília, Fundação Oswaldo Cruz, Brasília, DF, Brazil.

**Keywords:** occupational health, health facility planning, right to health, coronavirus infections

## Abstract

This theoretical-critical assay is intended to perform a detailed reflection on the mandatory payment of an additional compensation to health care professionals during the pandemic caused by COVID-19. For this consideration, academic and opinion articles, as well as national and state legislative proposals, were searched in dialogue with Regulatory Standard 15, which provides for unhealthy activities and operations. After reflection, the position taken is that payment is due for the duration of the pandemic, to all health care professionals working in the frontline against COVID-19.

## Introduction

Article 189 of the Consolidation of Labor Laws (Consolidação das Leis do Trabalho, CLT) conceptualizes unhealthy activities or operations as “those that, by their nature, conditions or working methods, expose employees to hazardous agents, above the tolerance limits established due to the nature and intensity of the agent and to the time of exposure to its effects,” as defined by Law no. 6,514/1977.^1^

In the new scenario of the pandemic caused by the severe acute respiratory syndrome coronavirus 2 (SARS-CoV-2), all individuals are potentially exposed to COVID-19, either in their workplace, in public transportation, or in stores and markets. Exposure to contagious diseases such as COVID-19, qualified as a biological agent, pursuant to Regulatory Standard 15 (Norma Regulamentadora 15, NR-15), is a triggering factor for the payment of additional compensation.

NR-15 defines the activities or operations deemed unhealthy and also stipulates the mandatory payment of the so-call additional compensation to all individuals who perform some work in the mentioned conditions, with compensation being levied upon 40, 20 or 10% of the regional minimum wage, depending on the degree of risk to which the professional is exposed (maximum, medium, or minimum, respectively) (Table 1).

**Table 1:** Synthesis of Regulatory Standard 15 (Norma Regulamentadora 15, NR-15)

Items	Description
1.5.1/15.1.1/15.1.2/15.1.3/15.1.4/15.1.5	Unhealthy activities or operations are those that are developed above the tolerance limits provided in Appendices no. 1, 2, 3, 5, 11 and 12 (Revoked by MTE Ordinance no. 3,751/1990), as well as those mentioned in Appendices no. 6, 13 and 14. Confirmed by means of an inspection report of the workplace for activities mentioned in Appendices no. 7, 8, 9 and 10. For the purposes of this standard, “Tolerance Limit” is understood as the maximum or minimum concentration or intensity, related to the nature and time of exposure to the agent, which will not harm workers’ health during their working life.
15.2/15.2.1/15.2.2/15.2.3	The performance of working in unhealthy conditions, pursuant to the subitems of the previous item, ensures workers’ right to receive compensation, which is levied upon the regional minimum wage, corresponding to 40% (forty per cent) for high-grade unhealthiness; 20% (twenty per cent) for medium-grade unhealthiness; and 10% (ten per cent) for low-grade unhealthiness.
15.4.1/15.4.1.1/15.4.1.2	The elimination or neutralization of unhealthiness should occur: a) with the adoption of general measures to maintain the work environment within the tolerance limits; b) with the use of personal protective equipment. Once unhealthiness is confirmed by a technical report from a dully qualified safety occupational engineer or an occupational health physician, the regional authority competent in matters of occupational safety and health is responsible to establish the additional compensation due to employees exposed to unhealthiness when its elimination or neutralization is unfeasible. The elimination or neutralization of exposed to unhealthiness will be characterized through expert assessment by a relevant agency confirming the absence of occupational health risks.

Source: Developed based on the Regulatory Standard 15 (Norma Regulamentadora 15, NR-15).

However, item 15.4 of NR-15 emphasizes that “elimination or neutralization of unhealthiness will determine the termination of compensation benefits,” which should occur after two supplementary measures: (i) “adoption of general measures” to maintain the “work environment within the tolerance limits” and (ii) “use of personal protective equipment (PPE)” (Table 1).

Such legal possibility may explain why PPE is the solution most adopted by employers to eliminate or neutralize risks, despite the guidelines provided by the International Labour Office.^2,3^ This organization establishes that risks to workers’ health and safety should be continuously identified and assessed, and that protection and prevention measures should be implemented according to a scale of priorities: (i) eliminating risks/hazards; (ii) controlling sources of risk/hazard using engineering/management techniques; (iii) minimizing risk/hazards through safety system projects, including management controls; and (iv) providing appropriate PPE by employers when risks/hazards cannot be prevented or controlled by collective measures.

The present theoretical-critical assay^4^ conducts a positioned reflection on the mandatory payment of additional compensation to health care workers in times of COVID-19 pandemic. In order to provide a theoretical support in dialog with the field, academic articles, opinion articles, and legislative proposals were searched.

### The pandemic context and the raised problem

In times of COVID-19 pandemic, it is easily perceivable that people are exposed to the virus in the several environments through which they move, from home to work, in addition to commuting to work, regardless of the mean of transport used. Therefore, the occupational field is essential in the creation of a strategy to face the pandemic,^5^ and health care workers are the most valuable and crucial resource in all countries.^6^

This importance is illustrated by the situation of Singapore, for example, where 68% of the 25 initial locally transmitted cases were related to occupational exposure.^7^ In Brazil, the second reported death due to COVID-19 was that of a domestic worker in Rio de Janeiro who was contaminated at work.^8^ In China, the first deaths were reported in Wuhan among seafood market workers.^9^

Furthermore, there is the fact that previously activities or operations that were previously safe became unhealthy, with high rates of contamination by SARS-CoV-2. This contamination results from numerous factors, such as high human crowding in the workplace, direct or indirect interaction with other individuals, and the free transit of individuals in these workplaces.^10^ Therefore, there is a great risk of viral transmission in workplaces, considering the possibility that those who go through these places may be infected, regardless of their awareness of their health status with regard to the infection.

In the case of health care professionals working in health care institutions, some categories have already entitled to additional compensation, but there is an undeniable increased exposure to the biological agent SARS-CoV-2 among these professionals,^11^ resulting from the performance of their work duties. Tal finding led to the following question: is additional compensation of 40% mandatory for health care workers who are in the frontline against the pandemic?

A similar question (applicable to municipal civil servants in the state of São Paulo, Brazil) was the object of a consultation request by the Council of Municipal Health Secretaries of the State of São Paulo (Conselho de Secretários Municipais da Saúde do Estado de São Paulo, COSEMS-SP) to Institute of Applied Health Law (Instituto de Direito Sanitário Aplicado, IDISA),^12^ and was also present in some opinion articles.^13,14^

Furthermore, such controversy about health care professionals was the object of three regulations (Normative Instruction [Instrução Normativa, IN]), pursuant to article 224 of the Rules of Procedures of the Federal Senate, namely: (i) IN no. 24/2020, authored by senator Romário;^15^ (ii) IN no. 30/2020, by Rose de Freitas;^16^ and (iii) IN no. 31/2020, also authored by Rose de Freitas.^17^ The issue was also contemplated in Bill no. 1,802/2020, which is still pending and aims to stipulate the mandatory payment, for the duration of the pandemic, of a maximum additional compensation of 40% for health care workers at private institutions; those who already received it would start to receive the same maximum rate.

Senator Romário, author of the bill, highlighted that it is not possible to determine the payment to civil servants (federal government, states, and municipalities), because the Execute Branch is the only one with competence to legislate on this matter, as indeed provided in the Constitution.

Some concrete victories in the matter were obtained in some localities, such as the Federal District (Distrito Federal, DF), pursuant to Law no. 6,859/2020, which mandates the payment of maximum additional compensation to all health care workers, benefiting public and private employees who work with patients in the treatment of COVID-2019.^18^ The municipal civil servants of Campinas (state of São Paulo) working in the frontline against COVID-2019 also started to receive maximum additional compensation, due to an agreement between the Public Labor Prosecutor’s Office (Ministério Público do Trabalho, MPT), the Municipal Civil Servants’ Union of Campinas, and the Municipal Health Council.^19^

This also raises another question: is it fair that only some workers receive the additional compensation of 40%? Or should it be paid to all of them, since they are exposed to the same biological agent, fight against the same pandemic, and experience similar risks, considering the due proportions? Is it reasonable to have an inequality in the treatment granted to public and private workers, since they are exposed to the same risk agents (biological, chemical, and physical)?

### The supported position: mandatory additional compensation to health care professionals

Based on the CLT and on NR 15, it can be observed that exposure to COVID-2019 raise the hypothesis of exposure to a biological agent and is a triggering factor for additional compensation, with the compensation rate varying according to the degree of risk, as previously indicated, unless another rate within the legal limits is agreed or a law is issued stipulating a specific rate, as shown in the examples above.

Since SARS-CoV-2 is a biologic agent, reading appendix XIV of NR 15^20^ is essential to answer the question proposed in this essay: “[...] list of activities that involve biological agents whose unhealthiness is characterized by qualitative assessment. Maximum level of unhealthiness: work or operations in permanent contact with: - patients in isolation due to contagious diseases and with objects used by these patients and not previously sterilized; - flesh, glands, viscera, blood, bones, leather, hair, and excrements of animals with contagious diseases (carbunculosis, brucellosis, tuberculosis); - sewage (galleries and tanks); and - urban waste (collection and industrialization). Medium level of unhealthiness: works and operations in permanent contact with patients, animals, or contagious material, in: - hospitals, emergency services, wards, outpatient clinics, vaccination venues, and others facilities providing human health care (applicable only to individuals who have contact with patients and with objects used by these patients and not previously sterilized); - hospitals, outpatient clinics, vaccination venues, and other facilities providing animal care and treatment (applicable only to individuals who have contact with these animals); - contact with animals used in the preparation of antiserum, vaccines, and other products in laboratories; - clinical analysis and histopathology laboratories (applicable only to technical staff); - departments of autopsy, anatomy and anatomical histopathology (applicable only to technical staff); - cemeteries (body exhumation); - stables and liveries; and - remnants of deteriorated animals.”

There are also chemical and physical agents, which are addressed in other specific appendices. In the case of biological agents, the article presents the regulations that provide for the qualitative assessment of exposure to the agent, resulting in a maximum (40%) or medium (20%) additional compensation.

The appendix establishes the due value in the case of exposure to SARS-CoV-2, which accounts for 40%: “[...] maximum level of unhealthiness: work or operations in permanent contact with patients in isolation due to contagious diseases and with objects used by these patients and not previously sterilized,” since this virus causes a contagious disease, i.e., COVID-19.

However, one of the regulations taken from appendix XIV requires answering three questions in order to assess whether an activity is effectively unhealthy due to a biological agent: (i) “is there biological risk?;” (ii) “is contact with the agent permanent?;” and (iii) “is the activity mentioned in appendix XIV?”. The three questions should be positively answered for an individual to have right to additional compensation.

Based on these questions, would all health care professionals who treat cases of COVID-19 be entitled to receive the 40% additional compensation? Nurses working in the intensive care unit (ICU) certainly would, there is no doubt about it; however, with regard to pharmacists, is contact permanent? Yes, it is, due to the fact that they are in hospital; therefore, their workplace keeps them in permanent contact with other health care professionals who directly assist patients under treatment, although pharmacists do not have direct contact with the user and with the biological agent.

Therefore, all health care professionals who are working with cases of COVID-19, fighting against the pandemic, will have the right to additional compensation at the maximum level of 40%, to be levied upon the regional minimum wage, pursuant to law.

A different question is also presented based on the situation already mentioned at the beginning of the text, which could not be faced until now, i.e., the regulations of item 15.4 of NR 15 (Table 1), which provides the termination of compensation in case of elimination or neutralization of unhealthiness. Therefore, two measures are necessary: firstly, a general measure, in order to maintain the work environment within the tolerance limits, and secondly, the use of PPE. Hence, the following question may be raised: in these cases of adoption of general measures and provision of PPE, should public and private health care workers stop receiving additional compensation?

This does not seem to be the best understanding, let us see the reasons. Firstly, let us ponder about the first measure, and then about the second one. What are “general measures”? Cleaning and disinfection of hospitals, health units, and health centers? If yes, are not these measures always performed? If they are, some professionals, even in non-pandemic times, continue to receive additional compensation, because these general measures do not definitely cease unhealthiness in that environment.

With regard to PPE, one may ask: does it have a proven efficacy of 100% in preventing health care professionals to get infected, even if all types of PPE were available for use, remembering that many hospitals experienced PPE shortage for a long time?^21,22^ Moreover, even if they were, would it eliminate environmental unhealthiness? Definitely not. Although it is possible to prevent transmissibility or even kill the biological agent, numerous procedures are still required to try to overcome environmental unhealthiness, which is always present, despite efforts to circumvent and solve it. Finally, the use of PPE in hospital settings is already a routine procedure in health care professionals’ life, even before the pandemic; therefore, it may not justify termination of additional compensation.

Furthermore, it is necessary to consider work commuting, since workers may contract the virus during this journey. Therefore, since COVID-19 will be considered an occupational disease if contagion results from in-person work and/or work commuting,^23^ it is honest and fair to consider work commuting as another space-time in which health care professionals may get contaminated. This already consolidated understanding corroborates the position of mandatory payment of 40% additional compensation to these workers.

Therefore, even if the indeterminate legal concept of “adoption of general measures” is conceptualized and that public and private health care professionals use all types of PPE available, environmental unhealthiness does not cease. In times of the pandemic, it is legal, fair, and ethical to obey the law or the existing agreements so that health care professionals receive maximum additional compensation.

Last, but not least, the Brazilian Constitution itself supports the position taken in this assay, especially based on the provisions of article 200, item VIII, which provides that the Brazilian Unified Health System (Sistema Único de Saúde, SUS) is responsible to defending the work environment - that is, it has the role of “collaborating in the protection of the whole environment, including the one where work takes places,” by making it healthier.

Based on article 200, especially on item VIII, it can be observed that additional compensation is supported in the constitutional text, by establishing workers’ right to a healthy workplace. When it is not possible to provide a health environment or to definitely remove the professional from that situation, which would be the ideal solution,^24,25^ it is imperative to pay an additional consideration, due to workers’ exposure to a context that may compromise their health, well-being, and physical and mental integrity.

In addition to financial compensation, especially in pandemic times, it is necessary to ensure psychological follow-up to health care professionals,^26,27^ as it was done in China,^28^ since, as observed by Teixeira et al.,^29^ the main problem faced by health care workers worldwide is the “risk of contamination that has caused sick leave, illness, and death, in addition to significant psychological suffering, which is expressed in generalized anxiety disorder, sleep disorders, fear of getting sick and of contaminating coworkers and relatives.”

Therefore, it is essential to improve mental health services, in order to provide sustainable, efficient, and equitable care.^30^

## Final considerations

The action of health care workers is essential in fighting against COVID-19. It is necessary to pursue ways to ensure a healthy work environment, instead of one that promotes illness and death. Since it is not possible to avoid unhealthiness, the legally provided compensation shall be paid to all health care professionals working to fight against the pandemic.

The right to life, integrity, and health should be the goals to be pursued by governments and health institution managers, in addition to the respect of the laws applicable to these professionals, such as those related to unhealthiness. Improvement of working conditions of these professionals should be a constant concern, not only in pandemic times.

Despite performing some activity not provided in the appendix XVI of NR-15, these health care workers should have their coverage ensured. In addition to local legislations and to agreements, there is the need for a national legislation that contemplates all health care professionals working in the COVID-19 pandemic, so that they receive the 40% additional compensation as measure to respect their dignity.
